# Meteorological Table

**Published:** 1859-10

**Authors:** J. G. Westmoreland

**Affiliations:** Smithsonian Institution, Sept., 1859, at Atlanta, Ga.


### Meteorological Observations,

**Taken by J. G. Westmoreland, M. D., for the Smithsonian Institution, Sept., 1859, at Atlanta, Ga. Latitude 33 deg. 45 min.—Longitude 7 deg. 30 min.—Height above the Sea, 1050 feet.**

| Day of Mo. | BAROMETER. | | | THERMOMETER. | | | RAIN. | CLOUDS. | | | WIND. | | |
|---|---|---|---|---|---|---|---|---|---|---|---|---|---|
| | 7 A. M. | 2 P. M. | 9 P. M. | 7 A. M. | 2 P. M. | 8 P. M. | | 7 A. M. | 2 P. M. | 9 P. M. | 7 A. M. | 2 P. M. | 9 P. M. |
| 1 | 30.00 | 30.05 | 30.00 | 66 | 8 | 70 | | 5 | 5 | 0 | S | S | W |
| 2 | 30.00 | 30.00 | 30.00 | 62 | 78 | 60 | | 5 | 0 | 0 | W | W | W |
| 3 | 30.05 | 30.12 | 30.05 | 72 | 84 | 74 | | 0 | 0 | 0 | W | S W | S W |
| 4 | 30.08 | 30.10 | 30.05 | 72 | 78 | 68 | 1.25 | 5 | 0 | 10 | S W | W | W |
| 5 | 30.10 | 30.18 | 30.15 | 68 | 80 | 71 | | 0 | 0 | 0 | W | N W | N W |
| 6 | 30.20 | 30.25 | 30.20 | 68 | 78 | 68 | | 10 | 5 | 0 | S W | E | E |
| 7 | 30.20 | 30.25 | 30.20 | 62 | 76 | 68 | | 10 | 5 | 5 | E | E | E |
| 8 | 30.25 | 30.30 | 30.25 | 66 | 76 | 70 | | 5 | 5 | 5 | E | E | E |
| 9 | 30.25 | 30.30 | 30.25 | 62 | 76 | 70 | | 0 | 0 | 0 | E | E | E |
| 10 | 30.25 | 30.30 | 30.20 | 62 | 78 | 70 | | 0 | 5 | 0 | E | E | E |
| 11 | 30.18 | 30.15 | 30.05 | 66 | 82 | 74 | | 5 | 5 | 0 | S E | S | S |
| 12 | 29.95 | 29.95 | 29.90 | 70 | 80 | 60 | | 5 | 0 | 0 | S W | W | W |
| 13 | 29.82 | 29.93 | 29.90 | 60 | 78 | 68 | | 0 | 0 | 0 | W | W | W |
| 14 | 29.95 | 30.09 | 30.05 | 62 | 73 | 70 | | 0 | 0 | 0 | W | W | W |
| 15 | 30.03 | 30.08 | 30.05 | 62 | 70 | 64 | 1.50 | 5 | 10 | 10 | S W | E | E |
| 16 | 29.80 | 29.70 | 29.80 | 62 | 78 | 70 | 3.87 | 10 | 10 | 5 | E | E | N W |
| 17 | 29.93 | 30.05 | 30.00 | 64 | 80 | 70 | | 0 | 0 | 0 | W | W | W |
| 18 | 30.00 | 30.05 | 30.00 | 66 | 76 | 68 | | 0 | 0 | 0 | W | W | S W |
| 19 | 29.90 | 29.90 | 29.90 | 66 | 74 | 68 | 0.37 | 0 | 10 | 10 | S W | S W | S W |
| 20 | 29.77 | 29.75 | 29.75 | 58 | 68 | 58 | 0.25 | 5 | 5 | 5 | W | W | W |
| 21 | 29.77 | 29.80 | 29.80 | 56 | 68 | 58 | | 5 | 5 | 5 | W | W | W |
| 22 | 29.90 | 30.05 | 30.05 | 56 | 68 | 62 | | 5 | 5 | 0 | W | S W | W |
| 23 | 30.05 | 30 09 | 30.10 | 54 | 68 | 60 | | 0 | 0 | 0 | S | W | W |
| 24 | 30.00 | 30.09 | 30.08 | 52 | 72 | 68 | | 0 | 0 | 0 | S W | S W | W |
| 25 | 30.08 | 30.00 | 30.00 | 58 | 74 | 68 | | 0 | 0 | 0 | W | W | W |
| 26 | 29.97 | 30.08 | 30.05 | 58 | 76 | 68 | | 0 | 0 | 0 | W | W | W |
| 27 | 30.05 | 30.15 | 30.15 | 60 | 80 | 62 | | 0 | 0 | 0 | W | W | W |
| 28 | 30.15 | 30.15 | 30.15 | 68 | 84 | 72 | | 0 | 0 | 0 | W | W | W |
| 29 | 30.15 | 30.22 | 30.20 | 66 | 80 | 72 | | 0 | 5 | 5 | W | W | W |
| 30 | 30.20 | 30.22 | 30.20 | 66 | 80 | 70 | | 10 | 5 | 5 | E | E | E |

EXPLANATION OF TABLE.—0 Signifies Entire Clearness—5 Half Cloudy—10 Entire Cloudiness.

